# Integrating and extending cohort studies: lessons from the eXtending Treatments, Education and Networks in Depression (xTEND) study

**DOI:** 10.1186/1471-2288-13-122

**Published:** 2013-10-05

**Authors:** Joanne Allen, Kerry J Inder, Terry J Lewin, John R Attia, Frances J Kay-Lambkin, Amanda L Baker, Trevor Hazell, Brian J Kelly

**Affiliations:** 1 Centre for Translational Neuroscience and Mental Health, University of Newcastle and Hunter New England Health, Newcastle, NSW, Australia; 2 Hunter Medical Research Institute, Newcastle, NSW, Australia; 3 Centre for Clinical Epidemiology and Biostatistics, University of Newcastle and Hunter New England Health, Newcastle, NSW, Australia; 4 Hunter Institute of Mental Health, Newcastle, NSW, Australia; 5 National Drug and Alcohol Research Centre, University of New South Wales, Sydney, NSW, Australia

**Keywords:** Cohort studies, Remoteness, Mental health, Individual participant data analysis, Research methods

## Abstract

**Background:**

Epidemiologic studies often struggle to adequately represent populations and outcomes of interest. Differences in methodology, data analysis and research questions often mean that reviews and synthesis of the existing literature have significant limitations. The current paper details our experiences in combining individual participant data from two existing cohort studies to address questions about the influence of social factors on health outcomes within a representative sample of urban to remote areas of Australia. The eXtending Treatments, Education and Networks in Depression study involved pooling individual participant data from the Australian Rural Mental Health Study (T_0_ N = 2639) and the Hunter Community Study (T_0_ N = 3253) as well as conducting a common three-year follow-up phase (T_1_ N = 3513). Pooling these data extended the capacity of these studies by: enabling research questions of common interest to be addressed; facilitating the harmonization of baseline measures; permitting investigation of a range of psychosocial, physical and contextual factors over time; and contributing to the development and implementation of targeted interventions for persons experiencing depression and alcohol issues.

**Discussion:**

The current paper describes the rationale, challenges encountered, and solutions devised by a project aiming to maximise the benefits derived from existing cohort studies. We also highlight opportunities for such individual participant data analyses to assess common assumptions in research synthesis, such as measurement invariance, and opportunities for extending ongoing cohorts by conducting a common follow-up phase.

**Summary:**

Pooling individual participant data can be a worthwhile venture, particularly where adequate representation is beyond the scope of existing research, where the effects of interest are small though important, where events are of relatively low frequency or rarely observed, and where issues are of immediate regional or national interest. Benefits such as these can enhance the utility of existing projects and strengthen requests for further research funding.

## Background

Cohort studies are invaluable in informing a wide range of research questions and they play a critical part in observational research methods where randomization is impossible due to practical and ethical issues. However, a range of factors such as representativeness, generalizability, the lengthy time frame needed to achieve outcomes, attrition, and associated research costs, mean that projects often cannot address the evolving, or indeed original, research questions of interest.

Pooling data from published studies, national databases, and collaborations between existing cohorts, have been used extensively to address these issues. The potential for pooled data to support the synthesis of existing research is exemplified by the application of meta-analytic techniques, combining summary statistics from existing studies. However, the limitations of synthesizing summary data, and the merits of combining individual participant data across studies are increasingly acknowledged [1]. These latter methods have been considered the gold-standard in research synthesis [2] and even replace methods based on summary statistics [3]. Furthermore, the increased capacity for online storage and transmission of datasets, together with calls for greater transparency, mean that opportunities for combining datasets for such purposes will only grow in coming years.

**Figure 1:**
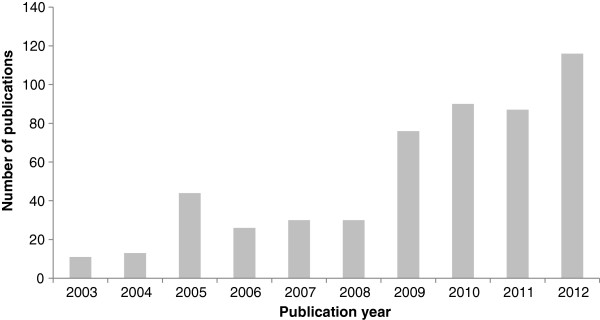
**Bar chart depicting increase in number of journal publications (articles or conference abstracts) including terms referring to pooling raw data, as found by a keyword search between dates 2003-2012 (N = 544).** [*Database*: *OvidSP titles listed 2003*-*2012*].

Known variously as ‘integrative data analysis’ [4], ‘mega-analysis’ [5], ‘cross-study analysis’ [6], ‘individual participant/patient data analysis’ [7,8] and even ‘individual participant/patient data meta-analysis’ [2,9-13], the combination of raw data from multiple studies into single analysis sets is increasingly undertaken. Figure 1 presents the frequency of publications using these terms over the past decade by year, which were identified by a keyword search of the OvidSP database for titles available between 2003-2012. It is evident that the use of these terms, and by proxy, associated analytical methods, has increased in recent years.

The potential for such research collaborations to maximise the benefits of cohort studies (e.g., identification of causal risk factors, retest effects, assessment of developmental trajectories), while minimising the negatives (e.g., cohort effects, participant fatigue, attrition, cost, length of time) [14] are clearly advantageous. Indeed, maximising the utility of existing resources is a key issue in many areas of research; for example, longitudinal cohort studies often address cross-sectional sub-questions to attract funding to support their ongoing activity (e.g., Dunedin Multidisciplinary Health and Development Study [15]). The potential to extend research through linkage to existing external data concerning the health of individuals and their environment is similarly acknowledged. Collaboration between research groups to address questions of common interest may present a worthwhile conduit for maximising the benefits to be derived from existing cohort studies, to sustain their ongoing activities and to support subsequent comprehensive investigations.

It has been recommended that new cohort studies employ commonly used measures from similar studies [16] and adopt standard protocols for assessment, measurement and statistical analysis [17] to ensure that some key benefits of combining data can be maximised. The combination of data from studies that have been planned to incorporate the potential for synthesis is known as prospective harmonization [18-20]. In contrast, retrospective harmonization refers to the synthesis of research studies that have been planned and conducted without this explicit intention in mind [18-20] and the benefits and weaknesses of these harmonization approaches have been previously discussed [18]. In both approaches consideration of the compatibility of studies in terms of design, as well as specific variables, is paramount. Access to detailed documentation regarding study protocols and outcomes are vital in assessing the capacity to validly combine datasets [18], which requires a high level of cooperation and communication between investigators.

There are several factors to examine when considering pooling individual participant data and the likely benefits of this undertaking. Table 1 lists a range of theoretical, statistical and practical rationale for pooling individual participant data, which arose from our own observations, as well as from existing discussion in the literature (e.g., [1,18,20]). While not exhaustive, or necessarily unique to pooled individual participant data analyses, these potential motivating factors are worthy of consideration by researchers considering combination of data across studies. The applicability of some of these arguments relate to the goals of the particular analysis. *Theoretical rationale* for pooling individual participant data across cohorts relates to the increased capacity of pooled data to address a given research question. *Statistical rationale* largely centre upon increasing sample size (e.g., increasing statistical power and detection of low frequency events). The *practical rationale* emphasize their capacity to maximise the benefits derived from existing resources.

**Table 1:** Reasons for combining experimental or observational research data across cohorts

**Potential rationale for combining data**	**Rationale for xTEND project**
**Theoretical**	
Pooling results in an area of research	**.**
Increasing generalizability	✓
Interest in known or potential sources of heterogeneity	✓
Replication of results	**.**
Questions of interest centre on association/modelling	✓
Interest in a subset of data	**.**
Identifying directions for future research	✓
**Statistical**	
Effects of interest are small but important	✓
More sophisticated models necessary	✓
Increasing observations of infrequent events	✓
Minimising effects of attrition over time	✓
Standardizing modelling used in predicting outcomes	**.**
Aggregation of data from repeated experiments	**.**
**Practical**	
Maximising existing resources	✓
Time efficiency/producing information of current public interest	✓
Cost efficiency	✓
Preliminary exploration to support funding for more comprehensive research design or inform later phases of research	✓
Features appealing to funding bodies	✓

Key benefits for the eXtending Treatment, Education and Networks in Depression (xTEND) study are denoted by ✓.

Several methodological aspects regarding the comparability of studies and variables assessed therein should also be given serious consideration. The suggested benefits of pooling individual participant data are made with the caveat that datasets meet *prima facie* conditions for the interpretability of combined data – that studies contain sufficient common information for analyses, and that their populations and methods are reasonably comparable. When pooling data from existing, independently conceived, cohort studies it is important to note that these studies were designed and implemented with a specific focus, which is not necessarily that of the combined analysis. While such threats to inference (i.e., sources of error and bias that prevent the meaningful integration and interpretation of combined data) are relevant to many scientific ventures, they are rarely addressable by traditional methods of research synthesis. Careful consideration of their implications is required for pooled individual participant data analyses [10]. Table 2 outlines some important potential threats to inference, which have long been recognised as relevant to inferential studies [21] and clinical research [22]. Recognition of instances where studies or measures are not comparable, or integration is undesirable for other reasons, may minimise the time and effort associated with undertaking pooled individual participant data analyses, as well as suggesting other methods of synthesis that are more appropriate, such as coordinated parallel analyses [17].

**Table 2:** Potential threats to inference when examining data across cohorts

**Threats**	**Description**
Contextual	The specific contexts from which samples were derived and recruited may influence results.
Historical	Events occurring between observations may influence results. May also relate to factors impacting on one cohort but not another at baseline assessments.
Time synchronicity	Studies are not conducted at a similar point in time, allowing a potential for factors or events associated with the time of administration to influence results. The length of time between follow-up assessments may also differ.
Geographic region	Similar to contextual factors, but specifically associated with features of geographical region.
Sampling frame and methods	Sampling frame (who was recruited) and methods could influence results (e.g., survey *vs*. phone based responses, and methods of following up non-responders).
Measurement equivalence	Measurement methods or characteristics may differ across cohorts or change differentially (e.g., for assessments to be comparable across samples and timepoints, we may need to examine participant responses and demonstrate that the same latent factors were assessed).

Finally, when pooling individual participant data across cohorts there are several other practical issues to be considered regarding material and personnel resources to undertake the task, the interests of the studies providing data, and ethical issues regarding access to and analysis of datasets. To aid others in their consideration of the benefits and pitfalls of individual participant data analysis, we examine some of the motivations and issues associated with pooling individual participant data, drawing upon our experiences with the eXtending Treatments, Education and Networks in Depression (xTEND) study [23].

### The xTEND study

Persons living in remote and very remote areas make up 2.3% of the Australian population [24] and approximately 85.7% of the country is classified as remote or very remote. Residents of these areas may be exposed to distinctive circumstances that impact on their physical and psychological health, including extreme environmental conditions, increased social isolation, and low levels of service accessibility. Of particular concern is evidence of increased suicide rates in rural compared to metropolitan populations, particularly among young men [25]. However, the representation of these populations in state and national surveys of health and wellbeing is severely limited [26-29]. Differences in the conceptualisation and measurement of remoteness between studies has also posed a significant challenge for the synthesis of literature regarding the influence of remoteness on health [30]. Thus, the generalizability of findings from urban studies about the determinants of wellbeing and the efficacy of health interventions for remote populations remain unknown.

As detailed elsewhere [23], the xTEND study aims to investigate the personal and social determinants of wellbeing in Australia and how these may be influenced by contextual factors associated with increased remoteness. The study is funded by several stakeholders, including the National Health and Medical Research Council, *beyondblue* - the Australian national depression initiative, and Hunter Medical Research Institute. To address these research questions access was required to a large, on-going cohort of participants who reflect the general population, reside in geographical areas that encompass urban, rural and remote areas of Australia, and among whom a broad range of physical and mental health attributes could be evaluated. Further, as these questions are of immediate regional and national interest, the timely delivery of research outcomes was considered essential.

Driven by awareness of the limitations of existing research on the health impacts of remoteness, and inspired by cross-national investigations into the influence of social and environmental factors on health outcomes, the xTEND study drew upon data from two existing independent longitudinal cohort studies based in New South Wales (NSW), Australia: the Hunter Community Study (HCS) [31] and the Australian Rural Mental Health Study (ARMHS) [30]. Many of the investigators on these studies were colleagues based in the same regional city and were broadly aware of the methods, scope and aims of each other’s research. The HCS collected self-reported psychosocial and physical data, along with clinical assessments, from a sample of persons aged 55 to 85 years who live in areas surrounding the major urban port of Newcastle, NSW (T_0_ N = 3253, 44.5% response rate, collected over two waves in 2004 and 2007; T_1_ N = 2252, 67.8% response rate, collected in 2010-2011). The ARMHS collected self-reported psychosocial and physical data from a sample of participants aged 18 years and older, oversampling from remote and very remote local government areas of NSW, to ensure adequate representation of these populations (T_0_ N = 2639, 27.3% response rate, collected in 2007-2009; T_1_ N = 1261, 47.8% response rate, collected in 2011-2012). Both studies recruited participants living in NSW from the Australian electoral roll and when combined provided a sample representative of the spectrum of metropolitan to remote communities, in largely contiguous local government areas. Baseline assessments in both studies included a range of social, physical and psychological indices. Further, access to participant data presented an opportunity to geocode participant remoteness in a consistent manner across cohorts. It was agreed that in aggregation these cohorts could provide a cost efficient platform to examine the research questions. At the point of inception of the xTEND project, both studies had collected baseline data and were due to begin design of 3-5 year follow-up surveys; consequently surveys used as part of the common follow-up were administered under the auspices of the parent studies.

### The current paper

The current paper outlines some of the advantages and challenges associated with combining individual participant data from multiple independent cohort studies. Drawing on the published literature, we discuss the benefits and pitfalls of individual participant data analysis and illustrate novel issues and applications encountered by the xTEND project. We address the theoretical and practical conditions for combining data as well as methodological considerations in the combination of data from multiple studies. While specific statistical methods used to address calibration of different measures of the same construct across studies have been detailed by others [1,19,32], we discuss general levels and methods of data comparability across studies, including some exploratory methods of calibration. Through our examples, we highlight some of the unique opportunities in extending collaborations by conducting a common follow-up phase. Apart from the obvious increase in assessment overlap, data from a common follow-up also facilitated the development and validation of statistical and psychometric approaches to the manipulation of non-identical measurements from earlier phases of data collection. Finally, we outline other practical issues regarding data access and management unique to individual participant data analysis and how these evolved and were addressed during the course of the xTEND project.

## Discussion

### A. Rationale for combining cohort data

Motivations for the initiation of the xTEND study, and those which may apply to other studies whose aims relate to the synthesis of existing research, are highlighted in Table 1. Analyses conducted for purposes of research synthesis (e.g., meta or mega analyses) derive somewhat different benefits from pooling data. These motivations are more comprehensively outlined elsewhere (e.g., [1,10]) and typically include: unified inclusion criteria; consistent definitions of variables and cut-points; and consistent statistical modelling and assessment of subgroups. Here we will focus on motivations for pooling data in studies addressing new or extended research questions, illustrated by some of the benefits achieved in the xTEND study. The four theoretical benefits identified in Table 1 are discussed first, followed by the four distinct statistical reasons for which increased sample size was a motivation for combining cohorts in the xTEND study, and finally we discuss the five practical motivations for the project.

#### A1. Theoretical rationale

##### Generalizability

**Figure 2:**
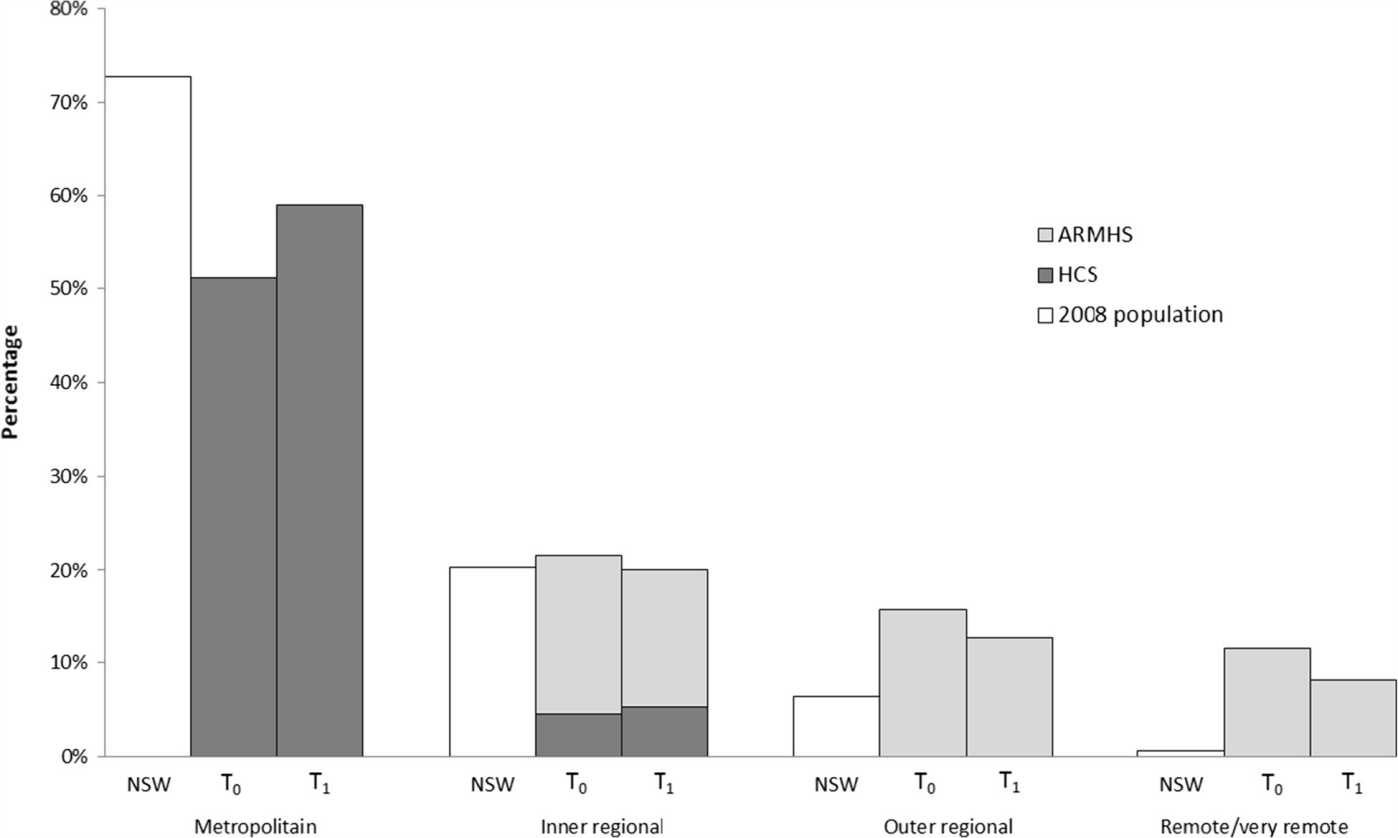
**Proportion of the pooled eXtending Treatment, Education and Networks in Depression (xTEND) sample at baseline (T**_**0**_**) and follow-up (T**_**1**_**) by remoteness category and Hunter Community Study (HCS)/Australian Rural Mental Health Study (ARMHS) membership, compared to New South Wales (NSW) population (2008).**

The importance of generalizability of study outcomes has been discussed at length in the literature. For cohort studies, limited representativeness may mean that results cannot be assumed to be true for unsurveyed or underrepresented subsets of the population of interest; this was a key incentive in devising the xTEND study. By pooling individual participant data from the HCS and ARMHS cohorts, the xTEND study accessed data over the spectrum of urban to very remote regions of NSW. Figure 2 presents a proportionate breakdown of the pooled xTEND sample by study membership, remoteness category and phase, demonstrating that in combination, these studies have the capacity to represent persons from across the spectrum of geographic remoteness in Australia.

##### Interest in known or potential sources of heterogeneity

Sources of heterogeneity refer to factors, either measured or unmeasured, that may influence differences within or between study outcomes. Cross-national studies allowing assessment of the influence of social policy and environment on health outcomes are perhaps the best known examples of such investigations. Curran and Hussong [4] provide a detailed discussion of some key sources and advantages of between-study heterogeneity (i.e., sampling, geographical regions, history, study design and assessment of measurement invariance/comparability). The opportunity to geocode participant remoteness in a consistent manner across the xTEND cohorts allowed us to examine the influence of geographical heterogeneity, not only on mental health outcomes but also its potential as an effect modifier. Other sources of heterogeneity not related to specific research questions (e.g., study inclusion/exclusion criteria) are perhaps better viewed as potential threats to inference, which need to be considered throughout the process of combining, analysing and interpreting combined data, and are discussed in section B (Methodological considerations).

##### Questions of interest centre on association/modelling rather than prevalence

The inferences that researchers wish to draw from combined data will often determine whether pooling individual data is a worthwhile undertaking. Samples are often compared with respect to prevalence rates (e.g., rates of diagnoses between the 1997 and 2007 Australian National Survey of Mental Health and Wellbeing [28,33,34]) and in these instances the challenges associated with pooling individual participant data may be superfluous. However, when patterns of association are of interest, such as the mediating effects of geographic remoteness on determinants of mental health outcomes, combining datasets may provide a more systematic and thorough investigation. Although differences in response profiles and rates may make comparisons of unpooled results untenable, it may still be valid to examine patterns of association across studies if appropriate factors associated with non-response are controlled for statistically. Thus, while simple comparisons of published data may be acceptable for questions about rates, prevalence and effect sizes, combining data may be more appropriate where the patterns of association between variables and outcomes are of major interest.

##### Identifying directions for future research

Areas for future research are typically identified based on gaps in current knowledge. However, where the published literature is unclear, pooled individual participant data analyses may assist in the identification of new research questions. Observational studies examining determinants of health outcomes within a longitudinal framework also present an opportunity to identify persons for whom health interventions may be desirable. The xTEND project sought to use baseline data from both cohorts to identify factors associated with alcohol, depression and suicidal ideation across a range of communities, with the aim of tailoring and piloting existing interventions for persons experiencing depression and alcohol issues: Self-Help for Alcohol/other Drug use and Depression (SHADE) [35]; and Partners in Depression project [36]. Responses to the common three year follow-up were used to identify persons who would be most likely to benefit from these interventions (e.g., those with current symptoms). Pooled analyses may also detect effects of interest for further exploration either within the individual cohorts (where constructs which are not assessed in common across cohorts could be additionally considered) or, where cohorts are on-going, by facilitating a common follow-up study.

#### A2. Statistical rationale: benefits of increased sample size

##### The effects of interest are small but important

Contextual effects on health outcomes, such as geographic remoteness, are typically small-moderate in size. However, the importance of even modest contextual effects in population research have become apparent, with increasing acknowledgement that even small shifts in the population distribution can have great impacts on population health outcomes [37,38].

##### The need to assess more sophisticated statistical models

An inability to examine interactions of geographic remoteness with other risk factors has potentially obscured the true influence of remoteness on health and wellbeing outcomes [39,40]; thus, our investigation facilitated sophisticated modelling of interactions, for which the parent studies were not originally designed (i.e., such effects may require greater statistical power or representation to detect).

##### Increasing observations of infrequent events

Low prevalence outcomes are infrequently reported in community cohorts. Hence, larger samples are required to obtain adequate data for the analysis of predictors and consequences of these outcomes. Suicidal attempts and ideation were outcomes of interest which are infrequently observed in community cohort samples. Increased observations of such cases mean that pooling individual participant data across studies provides greater ability in model estimation and increased power to fit more complicated models.

##### Minimising attrition effects

Cohort studies are vulnerable to the effects of attrition, reducing the available population from which inferences can be made and compromising the capacity to fit models with adequate statistical power and representativeness. While unlikely to address biases introduced by attrition (e.g., healthy persons may remain in cohorts longer), combining cohort data may provide one avenue from which to minimise the loss of power due to attrition. Differences in response rates between the current studies were partly due to differences in age ranges assessed and this difference was less marked in older groups, which were the focus of the xTEND project. While these cohorts were diminished in size at follow-up, in combination they remained representative of the population of interest (i.e., the spectrum of remoteness), enhancing our capacity for model replication and longitudinal inference over an extended period. Selecting appropriate research questions to investigate with pooled individual participant data are important in considering threats to inference (i.e., when considering questions of association, the influence of relative biases in sample recruitment may be quantified and are likely to be less of a threat to inference relative to studies describing prevalence).

#### A3. Practical rationale

When using existing data, results may be obtained in a time efficient manner compared to beginning a new study. Consequently, answers to research questions can be expedited, particularly where outcomes are of importance to the public, or preliminary results are required to leverage support for a larger study, or common follow-up study, which may be able to more fully address the questions of interest. Such results are relatively cost efficient, and can often be obtained without unnecessary duplication of tasks or additional burden on the target population. In the case of survey based cohort studies, reducing the necessity of identifying additional participants, and sending, receiving and processing survey data for analysis, may mean that the greatest costs associated with survey methods are eliminated. Resources can be refocused on the analyses and dissemination; the cost and technical resource considerations associated with pooling and analysing individual participant data across studies are noted later in this paper. These efficiencies, combined with the potential for pooled analyses to examine the feasibility of funding more comprehensive studies, are features that may be attractive to funding bodies. Additionally, support may be gained for sub-questions that are not the primary focus of the parent or pooled cohorts, but which may support the ongoing activities of the cohort, as was the case for the HCS in the xTEND study. Finally, findings from analyses of combined baseline data influenced decisions about the measures employed in a subsequent follow-up phase coordinated by the xTEND project, as well as informing ways to tailor interventions designed for implementation in these populations (e.g., Partners in Depression and SHADE interventions).

### B. Methodological considerations

Methodological considerations in the combination of data across cohorts focus on two general issues. Firstly, factors associated with cohort characteristics and the manner in which data were collected may influence the viability of inferences made using these data, particularly when inferences are derived using multiple cohorts. As mentioned, these potential ‘threats to inference’ are outlined in Table 2. Ways in which these factors were considered and examined in the xTEND project are provided, for example, under *B1* ‘*Features necessary for combining cohort data*’. Secondly, understanding conceptual and measurement overlap between cohort variables is necessary to enable valid statistical inferences to be drawn from combined cohort data. Acknowledging that few studies are likely to have a large number of directly comparable variables, in discussing *B2* ‘*Considerations in the analysis of combined data*’ we place particular focus on the degree to which variables overlap, illustrating such issues and how they may be examined and addressed using the xTEND example.

#### B1. Features necessary for combining cohort data

##### Contextual

Factors relating to the setting in which the sample was derived, including the physical and political realities, may influence study results. As detailed in our theoretical rationale, geographic remoteness was a contextual factor of key interest in the xTEND project. However, more broadly, such variation between studies could confound inferences drawn if it is not sufficiently quantified and taken into account. The context of participant recruitment may also influence the population attracted to the study (i.e., ARMHS participants agreed to take part in a study of mental health and wellbeing in rural Australia, while HCS participants were recruited to a study of wellbeing in older persons, with a particular focus on physical health). It is important to keep such factors in mind when interpreting results (e.g., rates of physical and psychological illness may vary across the parent studies due to differences in recruitment context and may not necessarily be attributable to the questions of interest). The influence of these characteristics on observed associations could be examined by assessing the moderating effect of cohort. As depicted in Figure 2, there was relatively little overlap in geographical representation between the ARMHS and HCS studies (i.e., primarily the ‘Inner Regional’ category). However, this overlap represents an important opportunity to explore potential confounding effects of cohort differences (i.e., examination of differences on the outcome of interest within this category by cohort when known cohort related factors are accounted for).

##### Historical

This potential threat typically involves differences in environmental exposures, which are thought to impact more upon longitudinal designs and intervention research in particular. While our parent cohorts could have been exposed to different historical events, both before and during the assessment period, it is unlikely that differences in exposure would have contributed to where they reside, which was a factor of interest in our analyses. For example, while state and federal elections were experienced by all within this combined cohort, they may have had different effects on urban and rural populations. However, this could be considered a facet of the effect of interest (i.e., geographic remoteness), as could, for example, differences in drought exposure or impact between remote communities and urban communities.

##### Time synchronicity

The majority of baseline data were collected within a six year period, meaning that there was some potential for baseline results to be confounded by factors associated with the time period in which they were conducted (i.e., secular effects). Events occurring during these periods were discussed by investigators, including a flood that impacted on the region in which the HCS was conducted during the second wave of baseline data collection. The common follow-up phase was synchronised, such that surveys were administered within a smaller one year window. Thus, events associated with responding were similar, and could be used to replicate results observed at baseline, to help assess the potential influence of any temporal threats to inference. An examination of the influence of time from baseline to follow-up will be an important consideration in any longitudinal analyses undertaken for this dataset, which may be controlled for, or which may necessitate the exclusion of the first wave of HCS participants who had a longer time between assessments (up to 6 years).

##### Comparable geographic regions

While the cohorts assessed in the xTEND study were from different geographic regions, they represented roughly contiguous local government areas. This minimised the possibility that the results were influenced by geographical factors other than those coded for in the analyses, such as participant remoteness.

##### Sampling frame and methods

This threat acknowledges that populations sampled, and the methods by which they are recruited and assessed, could result in systematic differences between cohorts, which could confound inferences drawn from analyses of combined data. However, such differences may be addressed to allow inferences to be drawn. Sampling methods were highly similar in the ARMHS and HCS cohorts, with participants selected randomly from the electoral roll, and while the ARMHS and HCS cohorts sampled different age ranges, there was significant overlap in the ages assessed, enabling the statistical modelling of selected age effects, at least among persons aged over 55 years. Comparisons of raw baseline response rates suggested these differed, though examination of documentation revealed samples displayed comparable rates of uncontactable or excluded persons (HCS 26.9% and ARMHS 25.2%).

##### Measurement equivalence/invariance

Studies comparing summary statistics across cohorts or the modifying effects of cohort on associations often assume invariance of the construct being assessed. However, even medical terms may differ in their meaning across cultures (e.g. Dyspnoea, in Dutch vs. US physicians) [41]. Pooled individual participant data presents a unique opportunity to test these assumptions and explore where aspects of invariance may occur and may aid in understanding the differences in the meaning of concepts between groups and reasons for the presence or absence of between group differences. Such techniques are crucial in several areas, notably when alternate language forms of a measure are used and verification of a common meaning is critical to understanding group differences and differences in association between groups. Although it would be excessive to assess the invariance of all measures across groups, the invariance properties of key concepts of interest should be considered [42]. In the xTEND study, the measurement and structural equivalence of the Assessment of Quality of Life-6D (AQoL-6D) scale [43] was assessed across cohorts and phases using nested multi-group confirmatory factor analyses. Confirming the measurement invariance of this construct allowed us to compare factors affecting quality of life across cohorts and timepoints, and provided an opportunity to contribute to the psychometric literature on this instrument [44].

#### B2. Considerations in the analysis of combined data

Combining variables for pooled individual participant data analyses is essentially a post-hoc process, which often requires its own design solutions. As differences in focus and methodology across cohorts are often reflected in the questions administered, as an initial step, it is important to identify any differences in study questions and response options and mechanisms (i.e., skip rules or instructions). Clearly, as noted by Bauer and Hussong, “*without common measures*, [*pooled individual participant data analysis*] *is a non*-*starter*” [16]. The process of pairing common measures for pooled analyses have been discussed elsewhere within categories of ‘stringent’ and ‘flexible’ harmonization [45], ‘complete’, ‘partial-proximate’, ‘partial-tentative’, ‘impossible’ harmonization [46] and ‘statistical’ harmonization [19]. Here we borrow the terminology of Curran and Hussong [4] to outline three broad circumstances under which data may be combined: ‘ideal’ circumstances, where data collected by cohorts is essentially the same; ‘less than ideal’ circumstances, where data are elicited using highly similar questions; and finally situations where statistical interventions are necessary to compare data across cohorts, noting ways in which such issues have been explored in the xTEND study and elsewhere. Combining datasets for pooled individual participant data analysis will often involve each of these three circumstances. Table 3 outlines some of the common variables within the parent studies linked by the xTEND project, and whether the combination of these measures was considered to be ideal (I), less than ideal (LI), requiring statistical intervention (S), and/or where measures of interest were missing from one cohort (M).

**Table 3:** **Comparability of Australian Rural Mental Health Study (ARMHS) and Hunter Community Study (HCS) measures**/**samples at baseline and common follow**-**up**

**Measure**	**Baseline**	**Follow-up**
**Demographics**		
Age	I	I
Gender	I	I
Education	LI	LI
Marital status	LI	LI
Retirement status	LI	LI
Employment status	LI	LI
**Social support**/**capital**		
Personal & network support	S	I
Sense of place (Environmental distress scale)	M	I
Family support	M	I
**Psychological distress**		
Kessler 10 (K-10)	I	I
Patient Health Questionnaire (PHQ-9) - Depression	M	I
Depressive symptomatology (CES-D)	M	I
Suicidal ideation	M	I
Solastalgia (Environmental distress scale)	I	I
Self-reported quality of life (AQoL-6D)	I^^^	I
Personal hopefulness (HOPES-12)	M	I
Neuroticism (Brief Eysenck scale)	M	I
**Physical illness and well being**		
Body Mass Index (BMI)	S	I
Chronic illness	LI	LI
Adverse life events	M	I
Alcohol use	M	I
Current smoking	LI	LI
Satisfaction with life	M	I
Physical and mental wellbeing (SF-36)	M	I
**Contextual effects **^#^		
Remoteness and SEIFA (postal code)	I	I
% rural employment, % land use for agriculture, and % population change (LGA)	I	I
Social capital and Health service accessibility (regional)	I	.

Note: *I,* Ideal circumstances for data combination; *LI,* Less than ideal circumstances for data combination (e.g., data re-coding required); *S,* Statistical intervention required for data combination; *M,* Missing from one sample or measures not comparable; ^^^ One subscale was missing from AQoL-6D at ARMHS baseline and imputation was required. ^#^ Contextual measures were derived using postal code information, from which indices at the relevant level of aggregation could be geocoded; *SEIFA*, Socio-Economic Indexes for Areas; *LGA*, Local government area.

##### Ideal circumstances

Where the same information or measures have been collected in comparable circumstances (e.g., same question and response options provided), data may be considered ideal for combination. This is not to say that we can necessarily assume that data may be combined without due care. Indeed, administering the same measures does not ensure that the same latent constructs have been assessed, either between or within constructs (see measurement equivalence). However, these data do lend themselves easily to the assessment of their comparability across studies. In addition to basic demographic indices, the ARMHS and HCS cohorts administered several standard measures of health and wellbeing (Table 3). Further, the opportunity for a common follow-up period substantially increased the number of common items that were assessed. While the Assessment of Quality of Life-6D was administered to both the HCS and ARMHS cohorts at baseline and follow-up, the measurement invariance of this instrument was assessed across groups and timepoints before data were combined or compared, to ensure that comparable latent constructs were assessed in these cohorts [44]; this was particularly important in light of the differing age profiles and research contexts for the parent studies.

##### Less than ideal circumstances

While ideal circumstances for data combination have been the mainstay of pooled individual participant data analysis, the benefits of ‘less than ideal’ circumstances are increasingly acknowledged for their potential to increase the number of studies included in an analysis [20]. Less than ideal data for combination are those that are elicited as responses to the same or similar questions but with slightly different wording or response options. These situations are often addressed by combining analogous options, to provide a new common variable, which is also known as ‘harmonizing’ data [16]. Although harmonizing data often has good face validity, care needs to be taken to assess the influence of harmonization on the generated variables and their associations with other variables. In the xTEND project, several variables, primarily demographic indices, were harmonized (e.g., educational status, marital status, retirement status, current smoking, self-reported chronic illnesses), which facilitated comparisons of their effects across urban to remote areas of NSW. Examples of harmonization processes have been detailed elsewhere (e.g., [45]).

##### Circumstances requiring statistical and design solutions

Where there is a desire to utilise instruments that are different, though conceptually similar, there may be opportunities to recalibrate these measures to provide comparable assessments of the same construct. Several good texts on equating measurement have been produced in the educational and psychological measurement literature (e.g., [47]). For example, the use of item response theory to create common metrics for assessment across studies is being recognized for its potential to maximise the utility of valuable cohort data in the psychological sciences [32]. Statistical standardization or centring has been cautiously applied to create a common scale for conceptually similar measures (i.e., where the same question is administered across cohorts, but a different number of Likert-style response options are provided) and applications and their limitations are discussed in more detail elsewhere (i.e., [19]). Where there are other known confounders in the administration of an assessment, it may be possible to apply corrections to address these biases. For example, in the xTEND study, there were potential biases associated with combining self-reported (ARMHS) and clinical (HCS) measures of height and weight across cohorts, which were to be used in calculating body mass index; these biases were substantially overcome by applying correction equations to the self-report data based on previous Australian research [48]. One preliminary method for assessing whether such harmonizing options are viable is to examine the associations that harmonized variables display with related predictors/outcomes in cohort subsamples in which it is believed these variables should display similar effects and determine whether cohort influenced the observed associations (i.e., [49]). Such statistical interventions are typically situation specific and contingent upon the type and amount of data available. Consequently, individual accounts of these processes may serve primarily as inspiration for addressing the particular situation with which researchers are faced.

The vast majority of statistical harmonization strategies in the literature call for at least some overlap in the items administered to assess a given construct. In the xTEND project, while both studies had assessed a range of common constructs, there were a relatively limited number of truly common variables at baseline. To address this, a common follow-up phase was conducted, which improved study overlap, as well as facilitating an examination of non-common baseline measures through triangulation (i.e., assessing phenomena or checking assumptions in multiple ways), by gauging the strength of association and common sensitivity of baseline measures by directly assessing their commonalities at follow-up. The following example is provided to illustrate some of the benefits of such measurement triangulation approaches. While the influence of social support was of particular interest to the xTEND project, it was not assessed using common items across cohorts at baseline. To facilitate the assessment of personal and network aspects of social support across the spectrum of remoteness assessed by the combined ARMHS and HCS cohorts, Allen et al. [50] recalibrated conceptually similar baseline measures of these constructs using data from a common follow-up administration of these measures in both cohorts. Several features were necessary to conduct this analysis: a common measure of psychological distress at baseline and follow-up; a follow-up period in which all relevant baseline measures were re-administered; an analysis of the baseline constructs or measurement elements commonly assessed by these two cohorts; and an analysis of the corresponding elements within the follow-up data, so that items assessing aspects of social support that were not commonly assessed at baseline could be omitted. Once the common items and concepts were identified, and their comparability assessed at follow-up, item scores could be standardized in the baseline cohorts to provide a common metric. This process of calibration through triangulation allowed us to explore the association of the calibrated social support indices with common psychological indices at baseline.

The common follow-up period provided an additional opportunity to impute baseline data that were not assessed by one cohort. Specifically, the xTEND project provided an opportunity to estimate missing ARMHS mental health subscale items from the AQoL-6D by facilitating access to follow-up data on these items from the ARMHS and to baseline and follow-up data from the HCS (in which the full AQoL-6D was administered) [44]. In essence, this situation is similar to that of planned missingness designs [51,52], wherein random sections of a cohort are asked subsets of questions for purposes of maximising the amount of information derived, while reducing survey length by imputing missing values based on the observed relationships. As the reason for missingness is known and can be coded for, the common follow-up allowed researchers to use imputation procedures to estimate the values of the omitted data, as the structure of the underlying correlation matrix can be derived to provide estimates of the associations between all model variables. These examples illustrate some of the key benefits of combining cohort data and, in particular, the benefits of conducting a common follow-up phase, where such an opportunity exists.

### C. Other considerations

#### C1. Resource considerations

While we have noted the cost and time efficiencies associated with the combination of data across cohorts, there are other resource issues that also need to be considered. When the combination of data from *several* studies is contemplated, there is likely to be a disproportionate increase in the logistic and resource considerations. The rigours of individual participant data meta-analysis in particular have been associated with a significant level of time, skill [53] and monetary [54] resources, although these requirements have decreased with modern technology [2]. Several researchers have noted that the task of pooling individual participant data is more expensive and time consuming than traditional meta-analyses [10] and, arguably, that this task is unjustified when the existing literature are adequately reported [55], although this is rarely the case in epidemiological synthesis. The pooling of particular cohorts for purposes other than research synthesis is likely to involve fewer datasets and more intensive collaboration with a smaller group of researchers, although it is unlikely to require less expertise in data management and analysis. Thus, the time, cost and personnel resources available, and the scope of the intended research questions, will be key factors that need to be considered before undertaking an analysis of pooled individual participant data from multiple cohorts. Importantly, the benefits of protocols and systems for systematically combining datasets, pairing variables and applying harmonization strategies have been explored previously [46]. Once early discussions of variables to be paired and harmonized between datasets had begun in the xTEND project, the development of automated tools for the generation of scripts applying discussed variable pairing and harmonization rules was undertaken so that these rules were applied in a consistent fashion that could be audited. Further, as these studies were ongoing, the capacity of this system to incorporate new and updated datasets as well as new phases of data collection were ensured.

#### C2. Maintaining the interests of existing studies

Pooled individual participant data analyses necessitate the collaboration and cooperation of research groups and opportunities for further collaboration may arise from the correspondence between cohort investigators [2]. However, the involvement of study participants and other stakeholders also needs to be acknowledged including any potential conflict arising from existing governance or ownership agreements. When funding for particular research questions are obtained through an essentially peripheral or independent source, such as in the xTEND study, it may be accepted that these questions can be satisfactorily addressed to varying degrees using the existing data across both cohorts. Nevertheless, when proposing a common follow-up, consideration also needs to be given to the original aims of the individual studies. Consideration of participant burden, associated with the administration of additional measures, consent procedures and the like, should be carefully evaluated. Indeed, even where areas of interest are common, there may need to be negotiation of how the interests of the parent studies will be maintained. Amongst other things, the xTEND project sought to corroborate the calibration of baseline instruments using a common follow-up. To reduce the redundancy across questions and the time burden on participants, a subsample of the ARMHS cohort received a survey containing some instruments used by HCS, which were not of interest to the study as a whole but would allow validation of the baseline data calibration procedures.

Finally, when dealing with two or more independent research groups, an important consideration will be reconciling the aims of these groups with the aims of the whole. In the case of the xTEND study, the original brief for the project included offering an intervention program (SHADE, Partners in Depression) for persons reporting symptoms of depression and alcohol use during the common follow-up. However, the HCS investigators objected to this component of the proposal, since it may have presented a historical threat to inferences derived from data collected during subsequent phases; that is, they wanted to preserve the naturalistic (non-intervention) elements of the longitudinal study. Thus, this component of the xTEND study was confined to ARMHS participants meeting criteria.

#### C3. Ethical issues

The ethical issues of combining datasets have rarely been discussed in the literature and as concluded by Cooper et al. “…*it remains an open question whether an individual*’*s agreement to participate in the original study also implies consent to have data included in a secondary analysis*. *Still*, *even this issue may be addressed simply by making data sets available to researchers only under the same rules of confidentiality that applied when the data was first collected*” [1]. However, this suggestion assumes that confidentiality is the participant’s only prerogative in deciding to participate in a research project. We would further argue that the reasons given for the project are integral to the participant’s decision to provide data and that the focus of the subsequent analyses (and indeed follow up) should not go beyond the general aims of the original projects. Indeed, this will likely hold true for the situation of individual participant data meta-analysis addressed by Cooper et al. [1], where data are combined for the purpose of synthesising studies on a single research question of interest. Thus, these ethical questions have also not arisen in response to traditional meta-analyses, as the questions answered by such studies are isolated to those for which they were originally collected and analysed. Similarly, both the ARMHS and HCS stated that data would be used to assess the determinants of physical and psychological wellbeing; and we decided that the additional consideration of the influence of remoteness on these determinants was not beyond the scope of this permission and informed consent. Our project went through an ethical review from the bodies that granted approvals to the original studies. However, it would be advisable when devising consent processes for new cohort studies that researchers include a specific item asking participants to indicate whether they consent to their de-identified data being used for broader purposes than those of the original study.

## Summary

The aim of the current paper was to provide an introduction to the potential benefits, as well as the challenges encountered and methods used, in the pooling of data from epidemiological cohorts, drawing on our experiences with the xTEND study. Many of the issues covered here are not unique to pooling individual participant data but are equally as pertinent, if rarely addressed, for drawing meaningful inferences from any combined data.

With each phase in the xTEND project, we are forced to consider new issues and challenges associated with combining datasets in this way – feedback from reviewers has been both challenging and encouraging. Accounts of the difficulties and solutions arising from other studies undertaking similar analyses have also been helpful (e.g., [32]). The xTEND study presented several methodological challenges within the context of studying how factors associated with mental health outcomes differed across the spectrum of remoteness. Notably, the measures initially administered within the parent studies varied considerably in terms of scope and metric. One of the specific goals of the xTEND project was to assess the influence of social connections on mental health across urban to very remote areas of Australia, by not only increasing the overlap between these studies at 3-year follow-up, but by facilitating baseline comparisons through harmonization and triangulation of important social indices. An additional element of the xTEND project, beyond the analysis of existing data, is that it has also facilitated an active collaboration between two ongoing cohort studies. This has maximised their future ability to inform the specific research questions of xTEND, along with validating the calibration of baseline measures, allowing analyses of longitudinal trends that would otherwise have been unmanageable.

### Lessons

In addition to sharing our research experiences and, hopefully, stimulating further discussion, there are several lessons from the current paper that are worth emphasising [with the related section heading provided]:

•When designing new cohort studies, consideration should be given to future opportunities for extending and synthesising research, by attending to guidelines aimed at facilitating such collaborations (e.g., [17]) [Background].

•Heterogeneity in study design may present a benefit and/or threat to pooling data across cohorts [Discussion A1 and B1];

•In addition to providing a more reliable way of synthesising research than aggregating published statistics, pooling cohort data may be of broader statistical benefit through increased sample size [Discussion A2];

•Combined data may present a time and resource efficient way of obtaining results [Discussion A3];

•Combined cohorts provide a mechanism for continuing existing cohort activities (e.g., facilitating supplementary questions; testing assumptions; initiating common follow-up phases) [Discussion A3];

•It is important to be mindful of the threats to inference associated with combining cohort data [Discussion B1 and Table 2];

•Where cohorts differ in their characteristics of interest, it is useful to have some overlap (e.g., in age distributions, geographical remoteness) so that the impact of these factors can be evaluated in analyses [Discussion B1];

•Where common constructs are measured and data pooling is being considered, it may be useful to identify whether these constructs are measured in ‘ideal’ or ‘less than ideal’ circumstances, or in ways that require statistical intervention (e.g., data harmonization, measurement triangulation) [Discussion B2];

•It is important to have some common constructs, measured in comparable ways, and preferably on multiple occasions - which permits cross-validation of findings and patterns of association, as well as an evaluation of the impact of the cohort on measurement (e.g., measurement invariance and stability) [Discussion B2];

•While utilizing existing data may have some efficiencies, the additional material, time and personnel requirements associated with combining data require close consideration [Discussion C1];

•The governance, procedural and scientific integrity of studies are important considerations, particularly where cohorts are on-going [Discussion C2]; and,

•Projects combining individual participant data for the purposes of new research questions should be mindful of the rights of research participants [Discussion C3].

## Abbreviations

ARMHS: Australian rural mental health study; HCS: Hunter community study; xTEND: eXtending treatments, education and networks in depression; SHADE: Self-help for alcohol/other drug use and depression.

## Competing interests

The authors declare that they have no competing interests.

## Authors’ contributions

JA and TJL prepared the current manuscript. KI, TJL, JRA, BJK, FK-L, AB, TH are investigators on the xTEND study and provided input regarding content and methods. All authors read and approved the final manuscript.

## Pre-publication history

The pre-publication history for this paper can be accessed here:

http://www.biomedcentral.com/1471-2288/13/122/prepub
